# *In Vitro* Human Keratinocyte Migration Rates Are Associated with SNPs in the *KRT1* Interval

**DOI:** 10.1371/journal.pone.0000697

**Published:** 2007-08-01

**Authors:** Heng Tao, Anthony J. Berno, David R. Cox, Kelly A. Frazer

**Affiliations:** Perlegen Sciences, Mountain View, California, United States of America; The Research Institute for Children, United States of America

## Abstract

Efforts to develop effective therapeutic treatments for promoting fast wound healing after injury to the epidermis are hindered by a lack of understanding of the factors involved. Re-epithelialization is an essential step of wound healing involving the migration of epidermal keratinocytes over the wound site. Here, we examine genetic variants in the keratin-1 (KRT1) locus for association with migration rates of human epidermal keratinocytes (HEK) isolated from different individuals. Although the role of intermediate filament genes, including KRT1, in wound activated keratinocytes is well established, this is the first study to examine if genetic variants in humans contribute to differences in the migration rates of these cells. Using an in vitro scratch wound assay we observe quantifiable variation in HEK migration rates in two independent sets of samples; 24 samples in the first set and 17 samples in the second set. We analyze genetic variants in the KRT1 interval and identify SNPs significantly associated with HEK migration rates in both samples sets. Additionally, we show in the first set of samples that the average migration rate of HEK cells homozygous for one common haplotype pattern in the KRT1 interval is significantly faster than that of HEK cells homozygous for a second common haplotype pattern. Our study demonstrates that genetic variants in the KRT1 interval contribute to quantifiable differences in the migration rates of keratinocytes isolated from different individuals. Furthermore we show that in vitro cell assays can successfully be used to deconstruct complex traits into simple biological model systems for genetic association studies.

## Introduction

**Figure 1:**
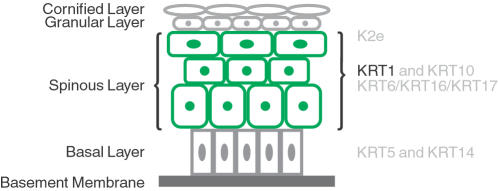
Expression of keratin genes in the stratified epithelium of the epidermis. As keratinocytes become post-mitotic and migrate through the epidermis from the basal into the spinous layer, *KRT1* and *KRT10* expression is up-regulated, replacing the expression of *KRT5* and *KRT14* [28], [29]. These expression changes are controlled at the transcriptional level [30], [31]. During terminal differentiation the keratinocytes migrate into the granular layer, and *KRT1* and *KRT10* are replaced by *K2e* [29]. During re-epithelialization in the wounded epidermis, cells preparing to migrate down-regulate *KRT1* and *KRT10* in favour of *KRT6*, *KRT16*, and *KRT17* [2], [3]. The repression of *KRT1* is thought to be necessary for normal terminal differentiation and migration [28], [29]. The figure is adapted from Porter and Lane [29].

When the outer covering of the skin, the epidermis, is injured a wound healing response is initiated. Wound healing is a complex process that occurs in different stages and involves many different cell types [1]. In epidermal keratinocytes undergoing activation at the wound edge the expression of intermediate filament genes is altered (Fig. 1). The expression of *keratin 6* (*KRT6*), *KRT16*, and *KRT17* is induced while the expression of *KRT1* and *KRT10* is down-regulated [2], [3]. These alterations in intermediate filament gene and protein regulation are thought to be necessary for the keratinocytes to make the morphological changes required for migration [2], [3].

Studies in the mouse show that inbred strains differ significantly in wound healing rates, indicating that in mammals there are genetic factors contributing to this trait [4], [5], [6], [7]. These results suggest that humans may also have DNA variants associated with wound healing rates. Because of the complexity of the wound healing trait a genetic analysis in humans would require a large number of individuals to identify such associations. However, using *in vitro* model systems of the various stages of wound healing, such as the re-epithelialization step, would enable the identification of genetic associations using considerably fewer individual samples.

In a previous study we determined that all 9 *KRT1* exons, as well as ∼22-kb of sequences upstream of the gene, are contained within a single 26-kb haplotype block located on chromosome 12 [8]. This 26-kb haplotype block contains 29 identified SNPs that resolve into three common haplotype patterns with frequencies greater than 10%. The two most common haplotype patterns, H1 and H2, occur at frequencies of 37.5% and 50% respectively in the population we previously examined for allele-specific expression differences of *KRT1*. In that study we demonstrated that the *KRT1* allele contained within haplotype pattern H2 is typically expressed at a greater than 8-fold higher level in human white blood cells then the *KRT1* allele contained within haplotype pattern H1 [8]. Based on the fact that down-regulation of *KRT1* in keratinocytes is important for the wounding response, any allele-specific differences in *KRT1* down-regulation in these cells may be reflected in migration rate differences.

Here we set out to determine whether genetic variants in the *KRT1* interval are associated with migration rates of human epidermal keratinocytes (HEK) in response to wounding. We performed a simple scratch wound assay [9], [10], [11] on keratinocyte samples isolated from different people and quantified migration rates, as measured by the speed of scratch wound closure. For each individual sample we genotype seven SNPs, which resolve all known *KRT1* haplotypes, thereby allowing us to examine most variants in the interval for association by proxy. Regression analysis identifies multiple SNPs in the *KRT1* haplotype block that are each associated with the HEK cell migration rate variation. Additionally, permutation analysis indicates that HEK cells homozygous for haplotype H1 migrate significantly faster than HEK cells homozygous for haplotype H2. Our results show that genetic variants in the *KRT1* interval are associated with the rate of HEK migration after wounding *in vitro*.

## Results

### Primary HEK cells

We examined cultured primary HEK cells derived from the epidermal layer of normal neonatal foreskin to determine whether they could be used as a model system to test our hypothesis that genetic variants in the *KRT1* interval are associated with variation in cell migration rates. To use the epidermal cells to test this hypothesis it would be necessary for them to express *KRT1* mRNA and translate it into protein at a sufficient level. Both mouse and human primary epidermal keratinocytes, including those derived from neonatal human foreskin, have been reported to express *KRT1* mRNA and protein when grown to confluence in permissive calcium concentrations (0.10–0.16 mM) [12], [13], [14]. To confirm this expression in the primary neonatal foreskin-derived HEK cells we cultured, we extracted mRNA from a total of six samples grown to confluence in a permissive calcium concentration, and used real-time PCR to measure *KRT1* mRNA. Using a method described by Vaitomaa et al [15], we calculated the *KRT1* mRNA copy number in the confluent HEK cells to be ∼30–60 copies per cell. Previous studies analyzing RNA metabolism in mammalian cells [16], [17], [18] have indicated that there are ∼10 mRNA species present at ∼10,000 copies per cell, 500 at ∼200 copies per cell, and 15,000 present at ∼10 copies per cell, in all. Thus, our calculation that *KRT1* mRNA is present at ∼30–60 copies per cell is consistent with it being expressed at a moderate level in the confluent primary HEK cells.

**Figure 2:**
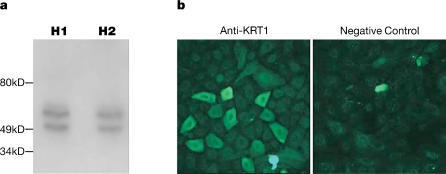
Characterization of KRT1 protein expression in HEK cells. a. Western blot analysis using anti-KRT1 antibody, with the left and right lanes containing proteins isolated from cells homozygous for the *KRT1* haplotype patterns H1 (sample 8 in Table 3) and H2 (sample 9 in Table 3) respectively. Replicate gels stained with Coomassie blue demonstrate equal levels of proteins loaded for the HEK samples. All six HEK samples examined contained similar levels of KRT1 protein. b. Immunohistochemistry showing distribution of KRT1 protein in two of the three confluent HEK samples examined. A subset of the anti-KRT1 cells displays strong membrane staining, while the remaining cells display low to moderate level membrane staining. Staining of the nucleus is present at the same level in the anti-KRT1 and negative control cells. All three HEK samples examined display similar KRT1 expression patterns.

We next examined whether the primary HEK cells translate the *KRT1* mRNA to protein using Western blot analysis and immunocytochemistry. Western blot analysis on cells from six different individual samples showed that KRT1 protein was present in confluent HEK cells. As shown for two of these HEK samples in figure 2a, two bands were observed: the expected 67 kD KRT1 band [19], as well as an additional smaller band of unknown nature. It is interesting to note a similar doublet has been observed by others using an anti-KRT1 antibody of different origin [13]. Immunocytochemistry analysis of samples from three different individuals showed that KRT1 protein is not evenly distributed in confluent HEK cells: while all cells express the protein, the majority express it at a low level and a subset express it at a very high level (Fig. 2b). Our observation agrees with previous immunocytochemistry studies showing variable KRT1 protein expression in primary epidermal keratinocytes cultured under similar conditions [12]. There were no differences in the distributions of KRT1 protein in the three HEK samples. Based on these data we concluded that *KRT1* is expressed and translated into protein in the primary neonatal foreskin-derived epidermal cells and thus they could be used as a model system to examine the genetic variants in the *KRT1* loci for association with variation in HEK migration rates.

### Assaying *in vitro* HEK cell migration rates

**Figure 3:**
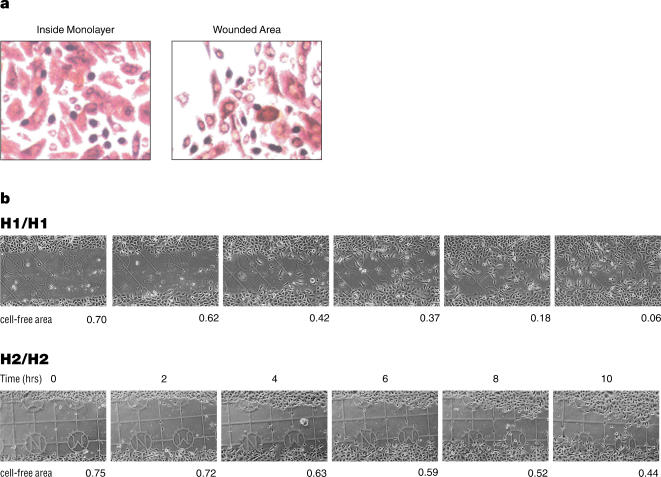
Wound assay. a. HEK cells were stained with BrdU five hours after wounding. The number of BrdU-positive cells observed at the wound area was roughly equal to the number observed within the monolayer (98±6), indicating that the two areas had approximately the same amount of cell proliferation. b. Migration of HEK cells in response to wounding. Digital photographs taken at the same positions at the time-points indicated show the migration of HEK cells into the cell-free areas of the artificial wound sites. Examples from cell-lines homozygous for *KRT1* haplotype patterns H1 and H2 are shown. The cell-free areas are given as a fraction of the 638 µm×956 µm area shown (the visible grid-lines are 175 µm apart). The cell-free areas from eight replicates are used to calculate the migration rate of each sample (see Methods).

To determine if there were quantifiable migration rate differences between different HEK samples in response to wounding, cell migration assays were performed as described [9], [10], [11] with slight modifications. The first set consisted of HEK samples derived from the epidermal layer of normal neonatal foreskin, isolated from 24 different individuals. These were grown to confluence on gridded coverslips. Scratch wounds were then created using a pipet tip and the wound site photographed digitally at two-hour intervals. Custom software was then used to calculate the cell-free area, and the cell migration rate was calculated using the changes in this area over time. A reproducibility test showed that repeated annotation of the same image produced an average difference of 1.2% of the total image area between replicates and that the difference never exceeded 4%. Cell proliferation assays showed that the increase of HEK cells within the wounded area was not due to increased proliferation (Fig 3a).

Exploratory analysis of the data suggested that the overall rate of HEK migration was most reliably represented by the change in the cell-free area between the 2 and 8 hour time points, as they represented a consistently linear region of the migration curve and provided the strongest agreement between replicate measurements of the same HEK sample. Therefore, for migration rate calculations we used the 2 and 8 hour time-points. As shown in Table 1 and Fig. 3b, we observed quantifiable differences in the median migration rates (from 8 replicates) of the 24 samples. We then obtained a second set of 17 additional samples, which also showed quantifiable migration rate differences when assayed in the same way.

**Table 1:** *KRT1* haplotype patterns in HEK cells.

	Haplotype sequence							Haplotype frequency (%)	
Haplotype pattern #	rs14024	rs597685	rs2741159	rs3759191	rs11170234	rs1567757	rs1567759	24 HEK (first set)	17 HEK (second set)
H1	G	T	T	T	G	C	C	45.8	29.4
H2	A	C	G	C	A	A	A	33.3	32.4
H3	A	C	T	T	G	C	C	18.8	20.6
H4	A	C	T	C	A	A	A	2.1	0.0
H5	A	C	T	C	A	A	C	0.0	8.8
H6	A	T	T	T	G	C	C	0.0	5.9
H7	A	T	T	C	G	A	A	0.0	2.9

### *KRT1* Haplotype Patterns in the HEK cells

To perform statistical tests to determine if the migration rate differences of the HEK cells are correlated with variants in the *KRT1* interval, we genotyped both sets of samples using seven SNPs that resolve all the haplotype patterns previously observed in the 26-kb haplotype block (Table 1) [8]. The SNP genotyping was performed after completion of the cell migration assays, in order to minimize subjective influence on the interpretation of the migration assay results. In the first set of HEK samples we observed four haplotype patterns, of which three are common with frequencies greater than 10% (Table 1). In the second set of HEK samples we observed six haplotype patterns, of which three are common and shared with the first set, and three are unique to this set of samples. In addition to differences in the minor haplotype patterns between the two sets of HEK samples, the relative frequencies of the two most common haplotypes, H1 and H2, differ (Table 1).

### Linear Regression: Association of SNPs with HEK Migration Rates

To test whether genetic variants in the *KRT1* interval are associated with migration rates of HEK in response to wounding we initially focused on the first set of 24 samples. We performed linear regression analysis examining each of the seven SNPs independently. The median migration rate, as the dependent variable, was regressed on the SNP genotype within each sample (coded 0, 1, or 2). *R* ^2^, the estimate of the percentage of variation explained by regression, was calculated as the ratio of regression sum of squares to total sum of squares. The results using the first set of samples demonstrate that two of the SNPs, rs14024 and rs597685, were significantly associated with HEK migration rate, while the other 5 SNPs showed no association (Table 2).

**Table 2:** Linear regression analysis of SNPs in the *KRT1* interval on HEK cell migration rate.

SNPs	HEK samples	*R* ^2^	*P* value
SNP rs14024	First set	0.182	0.037
	Second set	0.432	0.004
SNP rs597685	First set	0.182	0.037
	Second set	0.259	0.037
SNP rs2741159	First set	0.074	0.197
	Second set	0.439	0.004
SNP rs3759191	First set	0.055	0.269
	Second set	0.133	0.150
SNP rs11170234	First set	0.055	0.269
	Second set	0.076	0.284
SNP rs1567757	First set	0.055	0.269
	Second set	0.133	0.150
SNP rs1567759	First set	0.055	0.269
	Second set	0.37	0.010

We next examined the second set of 17 samples again analyzing each of the seven SNPs independently by linear regression analysis to determine if the association of genetic variants in the *KRT1* interval with HEK migration rate would replicate. Analysis of the data from the second set of 17 samples identified four SNPs significantly associated with HEK migration rate (Table 2). The association of SNPs rs14024 and rs597685 was repeated and SNPs rs2741159 and rs1567759 were also significantly associated in the second sample set. Interestingly, in the second sample set the SNP associations were more significant, and the *R* ^2^ values were substantially larger indicating that the *KRT1* interval accounted for a larger fraction of the variation in HEK migration rate, compared with the first sample set.

Although we examined each of the seven SNPs separately, the tests were not independent due to strong linkage disequilibrium (LD) in the *KRT1* interval. In the first sample set SNPs rs14024 and rs597685 are in perfect LD (*R* ^2^ = 1.0) and SNPs rs3759191, rs11170234, rs1567757, and rs1567759 are in perfect LD with each other. Thus, the association of SNPs rs14024 and rs597685 with HEK migration rate in the first sample set count as a single positive association. In the second sample set the number of observed *KRT1* haplotypes is greater (Table 1) and the LD in the interval is weaker than in the first sample set, with only SNPs rs3759191 and rs1567757 in perfect LD with each other. The four SNPs associated with HEK migration rate in the second sample set can be grouped into two LD bins, with rs14024 and rs597685 in one bin with moderate LD (*R* ^2^≤0.67) and rs2741159 and rs1567759 in another bin with strong LD (*R* ^2^≤0.88). However, the SNPs in the first and second bins, rs14024 and rs2741159, are only in weak LD (*R* ^2^≤0.23) with each other. These data suggest that there may be multiple independent genetic associations in the *KRT1* interval with HEK migration rate.

### Permutation analysis: Association of H1 and H2 with HEK Migration Rates

We next decided to analyze the two most common *KRT1* haplotype patterns, H1 and H2, which show allele-specific *KRT1* expression in human white blood cells [8], for association with HEK migration rate. By analyzing haplotype patterns we effectively assay most of the genetic variation in the *KRT1* interval, including any polymorphisms not yet identified. We focused on the first set of 24 samples comparing the migration rates of the five HEK samples homozygous for haplotype pattern H1 versus the five samples homozygous for the haplotype pattern H2. We found that the average migration rate was higher for the five HEK samples homozygous for H1 (15.067±3.052 µm/hr) than for the five HEK samples homozygous for H2 (11.442±1.6905 µm/hr) (the variation is expressed as ±half the average interquartile range, which is calculated as the difference between the 25^th^ and 75^th^ percentiles of the 8 replicates).

To determine the significance of these results, we calculated the likelihood of obtaining them under the null hypothesis of no correlation between the haplotype patterns and HEK migration rates, using a permutation test. Of the 24 samples in the first sample set, the five homozygous for H1 were classified as ‘homozygous H1’, the five homozygous for H2 were classified as ‘homozygous H2’, and the remaining 14 were classified as ‘other’ (Table 3). When we randomly shuffled the classification of the HEK samples into the three classes, only 4.5% of the 100,000 permutations performed resulted in a haplotype-associated difference in migration rate equal to or larger than the experimental observation, showing that the presence of *KRT1* haplotype patterns H1 or H2 is significantly correlated with quantifiable migration rate differences between the HEK samples (*P* = 0.045). We were unable to perform this test on the second set of 17 HEK samples, due to the fact that in this set there were only two samples homozygous for H2 and no samples homozygous for H1.

**Table 3:** *KRT1*-interval SNP^1^ genotypes, haplotype patterns and cell migration rates of the 41 HEK samples.

	SNP genotypes										
	rs14024	rs597685	rs2741159	rs3759191	rs11170234	rs1567757	rs1567759	Haplotype pattern #	Haplotype Class^2^	Median migration rate (µm/hr)^3^	Interquartile range^4^
**First sample set**											
1	AA	CC	GG	CC	AA	AA	AA	H2/H2	homozygous H2	10.5	3.5
2	AG	TC	TT	TT	GG	CC	CC	H1/H3	other	14.1	3.7
3	AA	CC	GG	CC	AA	AA	AA	H2/H2	homozygous H2	14.1	4.7
4	GG	TT	TT	TT	GG	CC	CC	H1/H1	homozygous H1	14.7	7.8
5	AG	TC	TT	TC	AG	AC	AC	H1/H4	other	16.4	6.0
6	AG	TC	TG	TC	AG	AC	AC	H1/H2	other	10.3	2.4
7	AA	CC	TT	TT	GG	CC	CC	H3/H3	other	13.9	9.1
8	GG	TT	TT	TT	GG	CC	CC	H1/H1	homozygous H1	19.5	8.6
9	AA	CC	GG	CC	AA	AA	AA	H2/H2	homozygous H2	10.2	2.6
10	AG	TC	TT	TT	GG	CC	CC	H1/H3	other	19.4	4.1
11	AG	TC	TT	TT	GG	CC	CC	H1/H3	other	10.4	6.7
12	AG	TC	TT	TT	GG	CC	CC	H1/H3	other	15.6	4.3
13	AG	TC	TT	TT	GG	CC	CC	H1/H3	other	7.82	2.13
14	AG	TC	TG	TC	AG	AC	AC	H1/H2	other	17.5	4.9
15	AA	CC	GG	CC	AA	AA	AA	H2/H2	homozygous H2	13.2	3.2
16	AG	TC	TG	TC	AG	AC	AC	H1/H2	other	16.9	3.8
17	AG	TC	TT	TT	GG	CC	CC	H1/H3	other	11.5	1.4
18	AA	CC	GG	CC	AA	AA	AA	H2/H2	homozygous H2	9.14	2.92
19	AG	TC	TG	TC	AG	AC	AC	H1/H2	other	14.9	3.8
20	AA	CC	TG	TC	AG	AC	AC	H2/H3	other	6.06	1.60
21	GG	TT	TT	TT	GG	CC	CC	H1/H1	homozygous H1	14.0	7.7
22	GG	TT	TT	TT	GG	CC	CC	H1/H1	homozygous H1	13.8	2.7
23	AG	TC	TG	TC	AG	AC	AC	H1/H2	other	19.2	5.9
24	GG	TT	TT	TT	GG	CC	CC	H1/H1	homozygous H1	13.3	3.9
**Second sample set**											
25	AG	TC	TG	TC	AG	AC	AC	H1/H2	other	19.5	4.0
26	AA	TC	TG	TC	AG	AC	AC	H2/H6	other	13.8	4.0
27	AG	TT	TT	TC	GG	AC	AC	H1/H7	other	21.2	4.8
28	AA	TC	TG	TC	AG	AC	AC	H2/H6	other	14.5	6.3
29	AG	TC	TT	TC	AG	AC	CC	H1/H5	other	27.2	3.7
30	AA	CC	TG	TC	AG	AC	AC	H2/H3	other	18.2	3.7
31	AA	CC	GG	CC	AA	AA	AA	H2/H2	homozygous H2	8.19	5.10
32	AG	TC	TG	TC	AG	AC	AC	H1/H2	other	17.7	5.2
33	AG	TC	TT	TC	AG	AC	CC	H1/H5	other	23.7	4.0
34	AG	TC	TT	TT	GG	CC	CC	H1/H3	other	19.5	3.6
35	AA	CC	TG	CC	AA	AA	AC	H2/H5	other	13.9	4.1
36	AG	TC	TT	TT	GG	CC	CC	H1/H3	other	22.9	6.3
37	AG	TC	TT	TT	GG	CC	CC	H1/H3	other	19.0	4.3
38	AA	CC	TG	TC	AG	AC	AC	H2/H3	other	15.1	5.1
39	AA	CC	GG	CC	AA	AA	AA	H2/H2	homozygous H2	15.7	2.5
40	AG	TC	TT	TT	GG	CC	CC	H1/H3	other	15.5	4.3
41	AG	TC	TT	TT	GG	CC	CC	H1/H3	other	14.6	3.5

1 The seven SNPs assayed here resolve all the haplotypes previously observed [8].

2 For the permutation test, the cells were classified as being homozygous H1, homozygous H2, or other.

3 The migration rate for each sample was calculated as the distance travelled by the migrating cells per hour, over a 6-hour time-period (between 2 and 8 hours after wounding). The median rate for each sample was calculated from eight replicates.

4 The interquartile range is the difference between the 75^th^ and 25^th^ percentile values of the 8 replicates.

## Discussion

Using an *in vitro* scratch wound assay we demonstrate that the genetic variants in the *KRT1* haplotype block are significantly associated with differences in the migratory rates of wound-activated HEK cells. Two of the SNPs associated with HEK migration rates are in exonic sequences. SNP rs14024 encodes a nonsynonomous amino acid change from a lysine to arginine in exon 9 (codon no. 633) of KRT1. This amino acid is located away from the highly conserved α-helical rod domains in the center of the KRT1 protein and is positioned in the carboxy tail region, a highly variable region for proteins in the intermediate filament family [19], [20]. The change from lysine to arginine is a swap of two positively charged basic amino acids that is unlikely to change the character of the protein at this position. SNP rs1567759 encodes a nonsynonomous amino acid change from a glycine to a cysteine in exon 2 (codon number 220) of KRT1B [21], a relatively newly discovered and functionally uncharacterized gene which lies largely in the same haplotype block as *KRT1* [8]. This amino acid is located in a subsection of the α-helical 1B region of the KRT1B protein that is not evolutionarily conserved. For these reasons we believe neither of the two coding SNPs is likely to alter the function of KRT1 or KRT1B. The other two SNPs associated with HEK migration rates, rs597685 and rs2741159, are located within intronic sequences of the *KRT1* gene. Interestingly, SNP rs597685 has been shown previously to lie within a *cis*-regulatory interval of the *KRT1* gene and appears to act as a positive regulator of *KRT1* expression [8].

Although the SNPs associated with HEK migration rates are likely directly affecting or acting as a proxy for genetic variants affecting *KRT1* expression, it should be noted that *KRT1* occurs within a large cluster of type II keratin genes on human chromosome 12. Thus, it is a formal possibility that *KRT1B* or another one of the type II keratin genes on chromosome 12 may be wholly or partially responsible for the association of the SNP alleles in the *KRT1* haplotype block with HEK migration rates.

Our study shows that *in vitro* model systems can successfully be used to deconstruct complex traits into simple biological model systems for the purpose of performing genetic association studies with small numbers of individual samples. The number of samples required to reach statistical significance for genetic association studies using an *in vitro* model system is difficult to predict *a priori* and will vary depending on the experimental design. Here, statistically significant associations with SNPs in the *KRT1* interval were identified in both the first set of 24 HEK samples and the second set of 17 HEK samples suggesting that sample sizes ranging from 15 to 30 will be sufficient for at least some of the *in vitro* model systems.

We have previously shown that *KRT1* has extreme allele-specific expression differences in human white blood cells [8], [22], with the *KRT1* allele contained within the H2 haplotype expressed at a significantly higher level than the *KRT1* allele contained within the H1 haplotype [8], [22]. A recent study suggests that allele-specific expression differences for a number of genes observed in white blood cells can be associated with physiological relevance in other tissues [23]. In the current study, HEK cells containing two copies of the H2 haplotype migrate significantly slower than those containing two copies of the H1 haplotype. It is tempting to hypothesize that human keratinocytes homozygous for the low-expressing H1 allele would down-regulate expression of *KRT1* more quickly and thus migrate sooner in response to wounding than those homozygous for the high-expressing H2 allele. However, as shown in Fig. 2b, when HEK samples containing the two *KRT1* alleles were grown to confluence we were unable to detect differences in the protein levels of KRT1, and our methods do not allow measurement of KRT1 levels in the migrating keratinocytes at the wound edge, and thus further studies would be required to examine this possibility.

## Materials and Methods

### HEK cells

Primary HEK cells were obtained from Cell Applications, Inc. (San Diego, CA). The cells were cultured in keratinocyte growth medium (0.4% bovine pituitary extract, 0.125 ng/ml epidermal growth factor, 5 µg/ml human insulin, 10 µg/ml transferrin, 0.39 µg/ml epinephrine. 0.33 µg/ml hydrocortisone, 0.16 mM CaCl_2_, from Cell Applications, Inc., catalog number 131-500) in 5% CO_2_ at 37°C and then cryopreserved. The keratinocyte growth medium is serum-free and fully supplemented with growth factors and antibiotics and was designed to promote attachment, spreading and proliferation of human keratinocytes on tissue culture plates. The HEK cells were delivered in two sets, with 24 samples in the first set and 17 in the second set. According to information from Cell Applications Inc., the two sets of HEK cells were derived from different hospitals in the greater San Diego Area.

### Assaying *KRT1* mRNA expression in primary HEK

Six of the primary HEK samples (numbers 2, 5, 6, 10, 11, and 12 in Table 3) were grown to 100% confluence, and TRizol (Invitrogen, Carlsbad, CA) was used to collect the cells and isolate RNA according to the manufacturer's instructions. cDNA was generated by reverse transcriptase of the RNA using SuperscriptII RT (Invitrogen) in the presence of oligo dT, followed by RNAseH treatment to eliminate the RNA. To generate a template for use in creating a standard curve, we amplified from cDNA and gel-purified a 53 bp fragment of *KRT1*, using the following primers: 5′ GTGGCAGTTCCAGCGTGA 3′ and 5′ GCATCTGGTTACTCCGGA 3′. We quantified the DNA fragment using a spectrophotometer, made 10-fold serial dilutions, amplified them using real-time PCR, and generated a standard curve for quantification of *KRT1* mRNA levels. We then performed real-time PCR using cDNA synthesized from 200 ng of total RNA from each HEK sample as template. We assayed the exonic SNP rs14024 using the same real-time PCR method as that described below for genotyping HEK samples. We estimated the number of copies of *KRT1* mRNA per cell by comparing the cycle threshold (Ct) values observed from each sample with those in the standard curve, using an estimate of 10–20 pg of total RNA for a typical mammalian cell [24], [25].

### Assaying KRT1 protein in the primary HEK samples

For Western blot analysis, HEK cells from sample numbers 8, 21, and 22 (homozygous for *KRT1* haplotype pattern H1) and numbers 9, 15, 18 (homozygous for *KRT1* haplotype H2) were grown in Lab-Tek chamber slides to confluency at 37°C in 5% CO_2_, in keratinocyte growth medium. The HEK cells were then lysed for 20 min at 4°C in lysis buffer (150 mM NaCl, 1 mM EDTA, 1% Triton X-100 and 50 mM Tris-HCl (pH 7.5)) and a protease inhibitor mix (Pierce Biotechnology). Protein concentrations were determined using a commercial Bradford Assay kit (Biorad). Proteins (20 µg) were separated on 8% SDS polyacrylamide gels, and transferred to polyvinylidene difluoride membranes. Membranes were blocked overnight with 5% nonfat dry milk in TBST (10 mM Tris-HCl, 0.15 M NaCl, and 0.05% Tween20, pH 8.0) and immunoblotted for 2 hours at room temperature with polyclonal goat anti-cytokeratin1 antibody (1∶500 dilution; Santa Cruz Biotechnology) followed by 1 hour at room temperature with horseradish peroxidase-conjugated rabbit anti-goat secondary antibody (1∶5000; Santa Cruz biotechnology). Western blots were developed with the ECL detection system (Pierce Biotechnology). To ensure equal loading of protein on the gel used for Western blot analysis, the identical protein aliquots from each sample were loaded on a duplicate gel. The duplicate gels were stained with Coomassie blue (0.2%) reagents, and equal loading was determined by visual inspection.

Immunocytochemistry was performed on the three HEK samples numbers 8, 9, 23. The cells were grown to confluence, washed three times with PBS, fixed in methanol for 10 min at −20°C, and blocked in PBS plus 0.05% Tween 20 and 5% BSA for an additional 10 min. The cells were then incubated for 1 hour with the same polyclonal goat anti-cytokeratin1 antibody as used for Western blot analysis, diluted 1∶200 in PBS plus 0.05% Tween 20, followed by 45 min with FITC-labeled rabbit anti-goat antibodies (Santa Cruz Biotechnology) diluted 1∶500 in PBS plus 0.05% Tween-20. As negative controls, cells were incubated with FITC-labeled rabbit anti-goat antibodies only. Both incubation steps were followed by three washings with PBS. Stained specimens were mounted in Permount mounting medium (Fisher Scientific) with coverslips. Cells were observed using a Zeiss LSM-410 laser scanning confocal microscope (Imaging Facility at Stanford University). Images were taken at 20× magnification.

### Assaying Cell Migration Rate after *In-Vitro* Wounding

The HEK samples were assayed in groups of four or eight, with all samples in a group coming from either the first or second set. Briefly, gridded coverslips (with 175 µm grids, Eppendorf AG, Germany) were placed on the bottom of each well in 24-well plates. Approximately 5×10^4^ HEK cells were plated in each well and grown to confluence. The cells were scratched once per well with a P20 pipette tip to create an artificial wound. Each sample was plated into eight wells, and thus had eight replicates of the wound assay. At 0, 2, 4, 6, 8 and 10 hours after wounding, plates were removed from the incubator and the wounded monolayers were washed three times with growth medium and photographed at the same location using the grid as a marker. Images of the 638 µm×956 µm areas were collected with a Nikon D70 digital camera coupled to the microscope (Fig. 3b).

A software user interface using C# and Microsoft Visual Studio was developed to analyze the digital images at the 0, 2, 4, 6, 8, and 10 hour time-points for the 8 replicates of each of the 41 HEK samples. The cell-free areas were measured as follows: each 638 µm×956 µm image was divided into a set of fifty vertical 19.1 µm strips running roughly perpendicular to the direction of the horizontal artificial wound. The extent of the cell-free area in each strip was defined as the region between the highest and lowest points at which a square section of that strip was completely free of cells. The cell-free area was traced manually along the wounded edge boundary and was calculated automatically.

### Calculation of Migration Rate

The cell migration rate was calculated as the distance travelled by the cells over time. Using the cell-free areas measured at the specified time-points, we calculated the change in cell-free area. Because the wound was made in the horizontal direction, the cells migrating into the previously cell-free area came from two directions, the top and bottom of the wound area. Thus, to calculate the distance travelled by the migrating cells we multiplied the change in the cell-free area (measured as a fraction of the total image area) by the height of the image (638 µm) and then divided by 2. The migration rate was then calculated as the distance (µm) travelled per hour.

### Cell Proliferation Assay

To check that the change in the cell-free area between the 2 and 8 hour time points was the result of HEK cell migration and not the result of cell division at the wounded edge, we performed cell proliferation assays (Fig. 3a). Monolayer HEK cells were wounded by scratching as described for the migration assay. Five hours after wounding, the cells were incubated with BrdU for 1 hour, and stained with the BrdU Staining Kit (Roche Diagnostics, CA) according to the manufacturer's instructions. Briefly, cells were incubated with BrdU for one hour, washed three times with PBS and fixed with ethanol. The cells were then incubated with anti-BrdU antibodies followed by anti-mouse-Ig-AP antibodies. Color was developed by incubating with nitroblue tetrazolium and X-phosphate. Nuclei of BrdU-positive cells were counted in 5 randomly chosen microscopic fields (0.5 nm^2^) at 400× magnification. Roughly equal numbers of BrdU-positive cells (98±6) were observed at the wound edge and in the intact monolayer away from the edge.

### Genotyping HEK samples

To genotype the 41 HEK cells we used real-time PCR. These assays were performed essentially as described by Germer et al [26] except that we used 10 ng genomic DNA, instead of cDNA, as template. The reaction conditions were as follows: 0.2 µM of each primers, 12 units of Stoffel Gold polymerase (PE Applied Biosystems), 10 mM Tris-HCl (pH 8.0), 40 mM KCl, 2 mM MgCl_2_, 50 µM each dATP, dCTP, and dGTP, 25 µM dTTP, 75 µM dUTP, 2 units UNG, 0.2× SYBR Green I (Molecular Probes), 2 µM ROX dye (Molecular Probes), 5% DMSO, and 2.5% glycerol, in a final volume of 50 µl. Each SNP was amplified with two forward allele-specific primers and a common reverse primer. The PCR reactions were performed on a GeneAmp 5700 Sequence Detection System (PE Applied Biosystems) as follows: 2 min at 50°C, 12 min at 95°C, then 45 cycles of 20 sec at 95°C and 20 sec at 58°C, and a final incubation for 20 min at 72°C. Flourescence data from kinetic PCR reactions were analyzed to calculate *C_t_* values for each PCR, as described [26].

The following unique primers were used: rs14024 allele 1 forward, TGGCAGTTCCAGCGTGAA, allele 2 forward, TGGCAGTTCCAGCGTGAG, reverse, GCATCTGGTTACTCCGGA; rs597685 allele 1 forward, GAACCCAGCATGATGGCTG, allele 2 forward, GAACCCAGCATGATGGCTA, reverse, ATCCCTGGTTCCTTTTGTGACT; rs2741159 allele 1 forward, TCTGAATCGTTGTGGGTTCA, allele 2 forward, TCTGAATCGTTGTGGGTTCC, reverse, CAACCAAGTTTATCTAAATGGAGAG; rs3759191 allele 1 forward, AAGGACCAGGTAACGTTACGT, allele 2 forward, AGGACCAGGTAACGTTACGC, reverse, GAAGGTAGGTTCACGTTCTCATG; rs11170234 allele 1 forward, TCACAGGTGCATCACCGGA, allele 2 forward, CACAGGTGCATCACCGGG, reverse, GGCAAAGGCTCCTCGCATCT; rs1567757 allele 1 forward, TTTACATGGACTCAGAACTAA, allele 2 forward, TTACATGGACTCAGAACTAC, reverse, GCAAGTTAAGCCATCACCTCA; rs1567759 allele 1 forward, TGCCTCCGCAGGTCACC, allele 2 forward, TGCCTCCGCAGGTCACA, reverse, AACTGGAACCAACAACCTGGA.

### Linkage Disequilibrium Analysis

We estimated two-site haplotype frequencies for the SNP pairs based on the observed genotypes in the second set of samples and the previously determined *KRT1* haplotypes (Table 1). We inferred *R* ^2^ from the estimated two-site haplotype frequencies using the equation: *R* ^2^ = (p11p22−p12p21)^2^/(p^+1^p^+2^p^1+^p^2+^) [27], where p^+1^ and p^1+^ are the minor-allele frequencies and p^+2^ and p^2+^ are the major-allele frequencies of the bi-allelic sites.
